# Feeding sites promoting wildlife-related tourism might highly expose the endangered Yunnan snub-nosed monkey (*Rhinopithecus bieti*) to parasite transmission

**DOI:** 10.1038/s41598-021-95166-5

**Published:** 2021-08-04

**Authors:** Eve Afonso, Rong Fu, Amaël Dupaix, Anne-Claude Goydadin, ZhongHua Yu, Cécile Callou, Petra Villette, Patrick Giraudoux, Li Li

**Affiliations:** 1 UMR CNRS 6249 Chrono-Environnement, Université Bourgogne Franche-Comté, 16 Route de Gray, 25030 Besancon, France; 2 Laboratory of Wildlife Management and Ecosystem Health, Yunnan University of Finance and Economics, Kunming, China; 3 Département de Biologie, ENS Lyon, 46 allée d’Italie, 69007 Lyon, France; 4 National Nature Reserve of BaiMa XueShan, Tacheng, China; 5 Archéozoologie, archeobotanique: societes, pratiques et environnements (AASPE), Muséum National d’Histoire Naturelle, CNRS, CP55, 57 rue Cuvier, 75005 Paris, France

**Keywords:** Conservation biology, Infectious diseases

## Abstract

An increasing number of studies have found that the implementation of feeding sites for wildlife-related tourism can affect animal health, behaviour and reproduction. Feeding sites can favour high densities, home range overlap, greater sedentary behaviour and increased interspecific contacts, all of which might promote parasite transmission. In the Yunnan snub-nosed monkey (*Rhinopithecus bieti*), human interventions via provisioning monkeys at specific feeding sites have led to the sub-structuring of a group into genetically differentiated sub-groups. The fed subgroup is located near human hamlets and interacts with domesticated animals. Using high-throughput sequencing, we investigated *Entamoeba* species diversity in a local host assemblage strongly influenced by provisioning for wildlife-related tourism. We identified 13 *Entamoeba* species or lineages in faeces of Yunnan snub-nosed monkeys, humans and domesticated animals (including pigs, cattle, and domestic chicken). In Yunnan snub-nosed monkeys, *Entamoeba* prevalence and OTU richness were higher in the fed than in the wild subgroup. *Entamoeba polecki* was found in monkeys, pigs and humans, suggesting that this parasite might circulates between the wild and domestic components of this local social–ecological system. The highest proportion of faeces positive for *Entamoeba* in monkeys geographically coincided with the presence of livestock and humans. These elements suggest that feeding sites might indirectly play a role on parasite transmission in the Yunnan snub-nosed monkey. The implementation of such sites should carefully consider the risk of creating hotspots of disease transmission, which should be prevented by maintaining a buffer zone between monkeys and livestock/humans. Regular screenings for pathogens in fed subgroup are necessary to monitor transmission risk in order to balance the economic development of human communities dependent on wildlife-related tourism, and the conservation of the endangered Yunnan snub-nosed monkey.

## Introduction

Human societies are living one of the most important paradoxes concerning ecosystems: while urbanization is increasing worldwide, interacting with nature is in ever-increasing demand^1^. Ecotourism is now considered to be one of the most thriving industries in the world and one of the most prominent cultural ecosystem services^2,3^. Besides providing these benefits, ecotourism is often an opportunity to promote educational programs that increase general audience awareness about ecosystem and endangered species conservation. However, protected areas developed for ecotourism are often mosaic habitats rather than wild landscapes. In other words, these may constitute agricultural, peri-urban, and fragmented areas in which the presence of humans and their activities would make them complex social–ecological systems^4,5^.

Wildlife-related tourism faces strong expectations from tourists to observe animals in their habitats; efforts to increase animal visibility are rarely compatible with the main behavioural characteristics of wild animals, i.e. mobility and evasiveness^6^. Facilities attracting tourists to ready-to-view and highly dense animal groups are often a great commercial success, especially in the case of animal species that are unusual and/or endangered^7,8^. A common way to concentrate animals at a given location is to habituate them to food provisioning. Some conservation programs have successfully implemented food provisioning to declining endangered wildlife populations that are food limited in order to increase their population size^9,10^. Supplemental feeding might act positively on survival, reproduction and body condition, which can maintain and at best increase population size^11^. However, a debate exists on the cost–benefit trade-offs of supplemental feeding of wildlife. Most studies focusing on food provisioning observed the modification of behavioural, physiological and ecological patterns of fed populations, as well as negative consequences for animal health^1,8,11,12^. Feeding sites have the potential to favour wild animal aggregations at high densities and/or home range overlap, greater sedentary behaviour and increased interspecific contacts, all of which might promote parasite transmission^11^. Therefore, food provisioning is expected to indirectly increase parasite transmission, especially when parasites have a density-dependent transmission^13^.

Human incursion into wildlife habitats creates various human–wildlife interfaces, including provisioning, research activities, hunting, and wildlife-related tourism^14^. The conversion of natural landscapes to agricultural or urban areas is recognized to increase zoonotic host diversity and to favour pathogen transmission, especially in mammal species^15^. Due to the close phylogenetic relationship between humans and non-human primates, bidirectional pathogen transmission can occur, and several studies have documented severe epidemics in the two host types^14,16^. Direct or indirect pathogen transmission at the human–primate interface has led to episodes of high mortality in many primate populations throughout the world, and is now considered to be one of the major threat in decreasing populations^16^.

Understanding the potential influence of feeding sites on parasite transmission in local social–ecological systems driven by ecotourism is an important step towards reconciling ecotourism and wildlife conservation. In such a context, we studied parasite exposure in a group of Yunnan snub-nosed monkeys (*Rhinopithecus bieti*). Non-human primates are a typical example of animals fed for ecotourism/wildlife-related tourism in many places throughout the world, and especially in Asia (see some examples in^17–20^). The Yunnan snub-nosed monkey is a species endemic to the Yunnan province of China, and is categorized as endangered on the IUCN Red list^21^. This species is threatened by environmental deterioration, accelerated deforestation, and poaching for food, medicinal and economic purposes^22^. Individuals of this species are now distributed among at least 15 discrete groups totalling less than 3000 animals^23^, in northwestern Yunnan and southeastern Tibet. It lives in high-altitude evergreen forests, between 2500 and 4600 m above sea level^24,25^. In the Baimaxueshan National Nature Reserve, rangers feed daily a small subgroup of habituated Yunnan snub-nosed monkeys with lichens, in several fixed feeding sites. This semi-captive subgroup is a support to reserve officers and local communities to increase public awareness about monkey conservation and promote ecotourism locally. However, human intervention to create a fed subgroup probably structured this group of monkeys, as the fed subgroup is genetically differentiated from the wild individuals living in surrounding areas, and shows lower genetic diversity^26^. Because the feeding sites are included in a mountainous agricultural landscape, livestock (mainly cattle and pigs) grazing areas overlap areas where fed monkeys live. This interface between domesticated and wild hosts has the potential to create hotspots of parasite transmission, with the risk to spread parasites in hosts living in direct or indirect contact with those hotspots.

To investigate the role of feeding sites on parasite transmission, we focused on protozoan parasites widespread in humans, domesticated animals, as well as non-human primates, i.e. *Entamoeba* species^27^. *Entamoeba* is a genus of intestinal protozoan parasites which primarily colonize the digestive system of a wide range of hosts in vertebrate and invertebrate species^28^. *Entamoeba* species have simple life cycles comprising a stage in the host intestines (the trophozoite), and a free-living form (the cyst) which can survive in the environment (soil and water) and be transmitted to a new host. Epidemiological studies of *Entamoeba* in non-human primates have mainly focused on captive animals in zoological parks^29–34^. Furthermore, relatively few studies performed on free-ranging non-human primates used molecular methods (PCR amplification of partial *18S rDNA*) to detect *Entamoeba* spp, and microscopy is not always suitable to differentiate all the known *Entamoeba* species, some of them being morphologically identical^35^. However, studies on free-ranging non-human primates converge to suggest that *Entamoeba* species could be highly prevalent in their faeces, with several *Entamoeba* species frequently co-occurring^27,35^. While non-human primates can be infected by host-restricted *Entamoeba* species (see a review in Elsheikha et al.^35^), they can also share *Entamoeba* species with livestock^36^ and humans^37^.

In this study, we collected faeces of Yunnan snub-nosed monkeys at feeding sites, as well as in surrounding mountainous areas. We screened feeding sites and the nearby village of Xiangguqing to sample faeces of domesticated animals and humans. After characterizing *Entamoeba* assemblages in the different hosts through high-throughput sequencing, we searched for determinants of parasite exposure in habituated monkeys. We addressed three key goals:

1. We determined if feeding sites overexpose fed monkeys to parasite, by measuring *Entamoeba* prevalence in fed and wild subgroups. We hypothesized that host aggregation at feeding stations might lead to higher prevalence in fed compared to wild monkeys. Little data are available to compare these two subgroups (e.g. age structures or demographic rates), nevertheless we have previously shown that fed individuals exhibit a deficit in heterozygotes and a mean relatedness two times higher in fed than in wild individuals^26^. Although highly debated, some examples suggest that a relationship exists between heterozygosity and some aspects of parasitism^38^. Inbred individuals might have higher chances to exhibit homozygosity for genes involved in disease resistance, and individual heterozygosity is thus expected to be a predictor of host susceptibility^39^. We thus hypothesized that monkeys with a low heterozygosity might have the highest probability to be positive for *Entamoeba*.
2. One of the most obvious strategies to mitigate the negative influence of feeding sites on parasite transmission is to space feeding stations more broadly, especially by avoiding domesticated animals and human settlements^11^. We thus determined if the distance from monkeys to other hosts might be used as a proxy for parasite exposure.
3. Extending our investigations to the hosts likely to frequent the feeding stations (mainly pigs and cattle), as well as those present in the nearest village (mainly domestic chickens and humans), we sought to determine whether different *Entamoeba* assemblage profiles co-existed in the different host. All or some of the hosts sharing the same *Entamoeba* parasites might have consequences both for the conservation of monkeys but also for human health and the health of domesticated animals. The fact that *Entamoeba* species are not highly host-specific makes this possible.

## Materials and methods

### Study area and sampling

**Figure 1:**
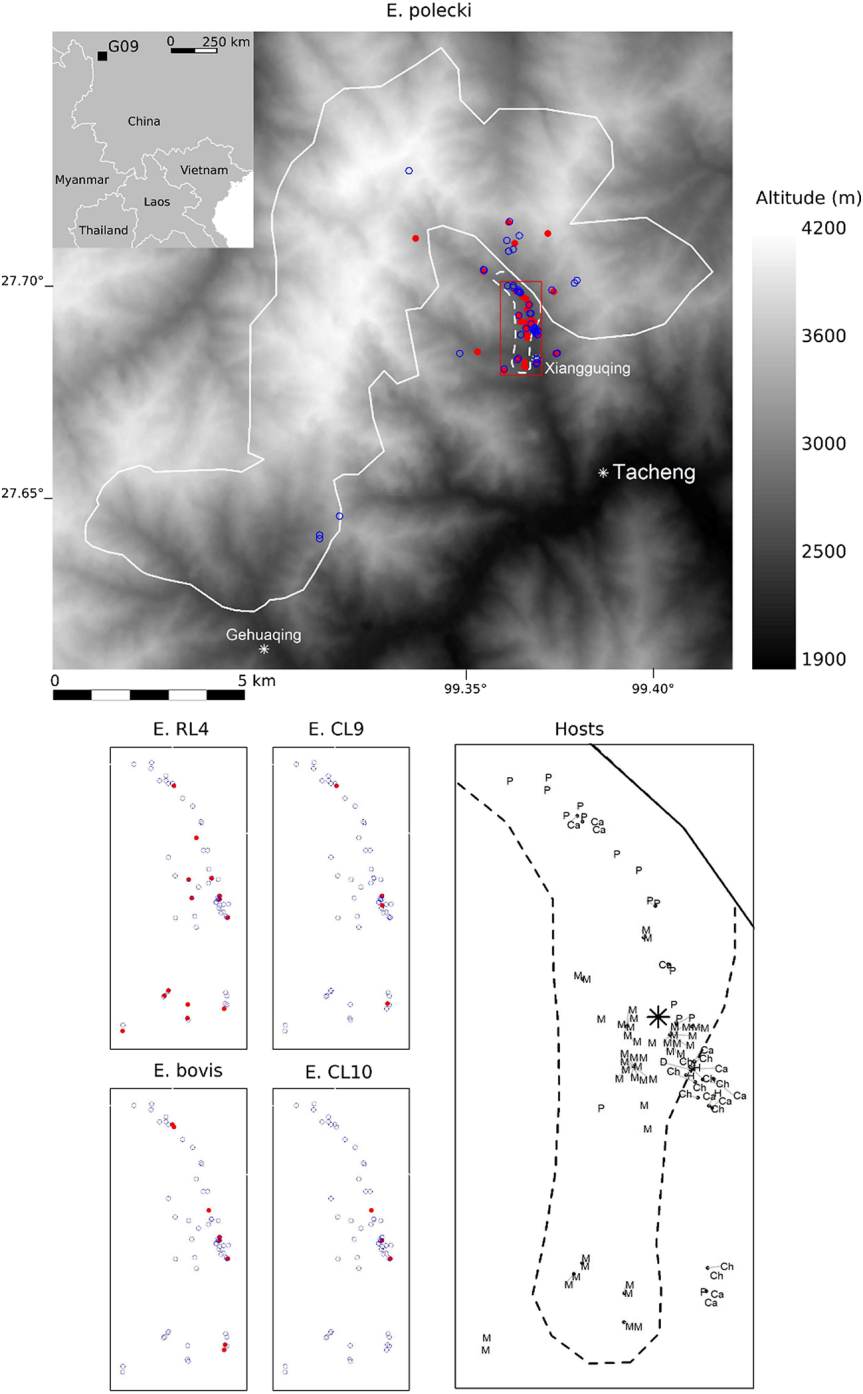
Location of the Xiangguqing/Gehuaqing group and distribution of host and *Entamoeba* species or lineages. Solid line, limit of the group after Wong et al. (2013), dotted line, limit of the area where monkeys are fed; Red solid circles, positive faeces; blue circles, negative faeces; star, non-monkey faeces centroid; M, monkey; Ca, Cattle; Ch, Chicken; D, dog; H, Human; P; pigs.

The study area covers about 82.9 km^2^ in the subtropical-temperate mountain Samage Forest (part of the Baimaxueshan National Nature Reserve) in the vicinity of Xiangguqing (响古箐) and Gehuaqing (格花箐) hamlets^23,40,41^, north-west of Tacheng (Fig. 1). Here, Yunnan snub-nosed monkeys form a large group that may comprise more than 900 individuals^23,42^. Reserve officers of the Baimaxueshan National Nature Reserve provision feeding sites located near Xiangguqing and Gehuaging hamlets with food (*Bryoria* sp. and *Usnea* sp. lichens, the natural staple food of this species, collected in the neighbourhood). They move the feeding sites a few hundred meters every two to three days to simulate the natural displacement of monkeys and to minimize the behavioural impacts of feeding. During feeding sessions, tourists can easily observe monkeys, but the reserve officers strongly limit potential physical contacts by maintaining a reasonable distance between the fed subgroup and tourists. Visitors are not allowed to give food to monkeys. The wild subgroup is quite elusive and distributed over the surrounding mountains.

Faeces of Yunnan snub-nosed monkeys were collected with the assistance of the reserve officers, from December 2016 to January 2017 and then from March to May 2017. Faeces from the fed subgroup were collected on the feeding sites after the feeding sessions to avoid disturbing monkeys. Faeces from the wild subgroup were collected opportunistically in the mountains while hiking on trails with the reserve officers^43^. We carefully collected only fresh faeces, relying on their general appearance. Faecal samples were georeferenced and stored in ethanol (70%) until laboratory analyses. A total of 91 faecal samples of Yunnan snub-nosed monkeys were collected in the field between December 2016 and May 2017. Using molecular genotyping, we determined that these samples corresponded to 44 distinct fed individuals and 30 distinct wild individuals (see details in Afonso et al.^26^).

Because a high *Entamoeba* spp. faecal prevalence was observed in Yunnan snub-nosed monkeys (see “Results” section), we went back to the field in May 2018 to collect faeces from domesticated animals and humans in and around the Xiangguqing hamlet, which is the nearest to the feeding sites. We visited every house, checking for the presence of pets and livestock (primarily pigs *Sus domesticus*, cattle *Bos taurus*, and domestic chickens *Gallus gallus*). One faecal sample was collected for each livestock species found in each house, coming from one individual or from latrines. Three human latrines were also sampled for faecal material. Livestock faeces were also collected directly on the ground in the feeding sites, with the assistance of the reserve officers. This systematic sampling lead to the collection of faecal samples in 16 pig groups, 11 individual cattle, 10 individual domestic chickens, one individual dog, and three human latrines. Due to the low sample size, the dog sampled in this study (PCR-negative for *Entamoeba*) was excluded from statistical analyses.

### Molecular analyses

To minimize potential contamination from the external surface of the faeces, we washed each sample (using ultra-pure water) before processing for DNA extraction. To collect faecal material for analysis, faeces were opened and 180–220 mg of stool was sampled from inside the faecal mass, avoiding the external surface. Total genomic DNA was extracted using the QIAamp Fast DNA Stool Mini Kit (Qiagen, Courtaboeuf, France) according to the manufacturer’s recommendations, after a step in 600 µl of lysis buffer (ASL, Qiagen) at 56 °C during 8–12 h in order to ensure a good homogenization of faecal material. Each sample was processed independently in an automated manner using the QIAcube platform (Qiagen). DNA concentration was then measured using a NanoVue Plus spectrophotometer (Biochrom). DNA extracts were stored at − 20 °C until DNA amplification.

Microsatellite genotyping of Yunnan snub-nosed monkeys is fully detailed in Afonso et al.^26^ and in the Supplementary Material S1. Individual genotypes were used to assign each faeces collected in the field to distinct individuals, then individual faecal prevalence of *Entamoeba* species were assessed. When genotyping revealed that several faeces originated from the same individual monkey, one faeces sample was randomly selected per individual for the subsequent analyses, after ensuring consistent results (positive/negative for *Entamoeba*) between replicates.

*Entamoeba* DNA was detected using the protocol developed by Vlčková et al.^44^. First, conventional PCR was applied to all samples. An approximatively 270 bp long region of *18S rDNA* was amplified using *Entamoeba*-specific primers 673f. (5′-ATYAGATACCGTCGTAGTCC-3′) and 942r (5′-GTWCGGTCTTGGTAAGTTTTC-3′) dual-indexed following a methodology adapted from Fadrosh et al.^11^ (see primers in Supplementary Material S2). The PCR mixture contained 1 × HotStarTaq Master Mix kit (Qiagen), 673f. (0.1 µM), 942r (0.1 µM), 0.5 mM of MgCl_2_ (final MgCl_2_ concentration in the reaction = 2 mM), DNA extract (10–50 ng), and PCR-grade water. The PCR program consisted of an activation step of 95 °C for 15 min, followed by 38 cycles of denaturation at 95 °C for 25 s, annealing at 55 °C for 30 s, and extension at 72 °C for 30 s. A final extension was performed at 72 °C during 5 min. The PCR products were separated and visualized using the QIAxcel device, and a QIAxcel DNA high-resolution kit (Qiagen).

PCR products of samples PCR-positive for *Entamoeba* DNA were pooled together, by regrouping separately amplicons from Yunnan snub-nosed monkey and amplicons from livestock and humans. Pooled amplicons purification was performed using a Pippin Prep (Sage Science, Massachussets, USA). Amplicon sequencing was performed using a MiSeq Reagent kit v2 (2 × 250pb paired-end reads) in the Illumina MiSeq Platform.

### Data processing

#### Bioinformatics

Read demultiplexing and primers trimming was performed using the Cutadapt software^45^. We then performed all subsequent analyses using R 3.5.1 software (R Core Team, 2018) and the packages Ape^46^, Biostrings^47^, Dada2^48^, Decipher^49^, Phangorn^50^, Phyloseq^51^, and ShortRead^52^.

We followed recommendations of Vlčková et al.^44^ and Galan et al.^53^ to filter sequences and perform denoising. Sequences with an expected number of sequencing errors of at least one were removed. We discarded one individual (a monkey) yielding fewer than an arbitrary threshold of 500 sequences^53^, supposing that a low number of sequences per sample might limit the completeness of *Entamoeba* assemblage detection^53,54^. After creating an operational taxonomic unit (OTU) table, we removed from each sample all OTUs that account for < 0.5% of overall sequences, supposing that these OTUs might be remaining chimera or incorrectly assigned sequences^53^. Finally, we removed sequences that were present in only a single sample^44^.

#### Taxonomic assignment and phylogenetic analyses

OTUs generated in this study were aligned with sequences from Stensvold et al.^55^ and Jacob et al.^28^, using the Decipher algorithm as implemented in the Decipher R package^49^. The phylogenetic analyses included 29 partial sequences of the *18S rDNA* gene in *Entamoeba* species. We tested which nucleotide substitution model was better suited to our sequence data by using the phangorn package in the R software^50^.

We used the terminology proposed by Stensvold et al.^55^ and Jacob et al.^28^ to describe *Entamoeba* taxonomic diversity: OTUs were assigned to an *Entamoeba* species when this species was previously described in terms of morphology and molecular data. Subtype (ST) is a genetic cluster within the range of diversity of a defined species, with sequence divergence within a ST being not greater than 3%. Ribosomal lineages (RL) corresponds to organisms for which ≥ 80% of the SSU rDNA gene has been sequenced and there is a divergence of ≥ 5% with known sequences, while conditional lineages fills the same criteria, except that < 80% of the SSU rDNA gene has been sequenced.

Taxonomic assignment of the OTUs generated in this study was then performed by matching BLASTn searches and phylogenetic analyses; sequences published in Stensvold et al.^55^ and Jacob et al.^28^ were used as a reference.

#### Statistical analyses

Because monkey faeces were collected during a period of six months, we tested for temporal autocorrelation in *Entamoeba* PCR-positivity using a Durbin–Watson test implemented in the R package lmtest. A faecal sample was considered positive for *Entamoeba* when at least one *Entamoeba* OTU was detected. Fed monkeys were expected to be more frequently positive for *Entamoeba* compared to wild monkeys, and we analysed separately the data for the two subgroups to avoid statistical confusion.

We determined if the faecal prevalence of *Entamoeba* OTUs or *Entamoeba* species/lineages differed among host types using Pearson's Chi-squared Test for Count Data, or Fisher's Exact Test for Count Data when data did not meet Cochran rules. Faeces samples from monkeys and other hosts were not collected during the same sampling session and were thus analysed separately to avoid incorrect interpretations due to inter-annual variations.

*Entamoeba polecki* was widespread in Yunnan snub-nosed monkeys (see results). Therefore, we used a logistic regression to link the logit of the probability of an individual to have a faecal sample PCR-positive for *E. polecki* (i.e. at least one of the four *E. polecki* OTU) to predictor variables: distance to livestock and humans, individual heterozygosity, and subgroup. Livestock distribution did not always coincided with human locations, and overlapped feeding sites, especially pigs and cattle. Distance to livestock and humans was then assessed for each monkey as the distance of one given faeces to the centroid of livestock and human sample locations. Individual heterozygosity in monkeys was approximated by the proportion of heterozygote loci over the 10 microsatellites used to determine individual genotypes (i.e. multilocus heterozygosity). Both distance to livestock and humans and individual heterozygosity in monkeys are confounded with the subgroups. The fed individuals are close to Xiangguqing, and they exhibit lower heterozygosities than wild individuals^26^. We thus permutated the order of the subgroup covariate among explanatory variables to proceed to model selection, in order to detect possible collinearity (see models tested Table 1). The models were compared using the Akaike’s Information Criterion, corrected for small sample size (AICc^56^). AICc differences between the best model and all other considered models (Δi = difference between AICc and the lowest AICc value) were calculated to determine the relative ranking of each possible model. The model with the lowest AICc represented the best compromise between the residual deviance and number of parameters^56^. When Δi < 2, the most parsimonious model (i.e., that with the fewest parameters) was selected.

**Table 1:** Binomial model comparisons of the probability to be PCR-positive for at least one *Entamoeba polecki* OTU in Yunnan snub-nosed monkeys, related to predictor variables.

Model	LL	K	n/K	AICc	Δ_i_	w_ic_
Distance + heterozygosity + subgroup	− 31.55	4	18.5	71.69	0.00	0.46
**Distance + heterozygosity**	− **32.92**	**3**	**24.67**	**72.18**	**0.49**	**0.36**
Distance + heterozygosity + subgroup + distance: heterozygosity	− 31.2	5	14.8	73.92	2.23	0.15
Distance	− 36.77	2	37.0	77.71	6.02	0.02
1	− 47.33	1	74.0	96.72	25.03	0.00
Subgroup + distance + heterozygosity	− 31.55	4	18.5	71.69	0.00	0.45
Subgroup + distance	− 33.39	3	24.67	73.13	1.44	0.22
Subgroup	− 34.67	2	37.0	73.51	1.83	0.18
Subgroup + distance + heterozygosity + distance: heterozygosity	− 31.52	5	14.8	73.92	2.23	0.15
1	− 47.33	1	74.0	96.72	25.03	0.00

LL, Maximized log-likelihood; K, Number of estimated parameters; n/K, number of observations/K; AICc, Akaike’s Information Criterion; Δi, difference between AIC and the lowest AIC value; wic, Akaike weight. The final selected model is in bold.

We then searched to determine if different *Entamoeba* assemblage profiles co-existed in the different host types. OTU richness (i.e. the number of OTUs per faecal sample) was compared among fed and wild monkeys using a Fisher exact test for count data. OTU diversity was compared among fed and wild monkeys using a Permutational Multivariate Analysis of Variance Using Distance Matrices (PERMANOVA), with 999 permutations and Bray–Curtis dissimilarities. Finally, a Principal Coordinate Analysis (PCoA) for Bray–Curtis dissimilarities was used to visualise among-sample differentiation in relative abundances of *Entmoeba* OTUs.

All statistical analyses were performed using the R software, using the packages Phyloseq and Vegan^57^.

### Ethics statement

Field sampling was carried out with the authorization of the Authority of the Baima Xueshan Natural Reserve. Because the present study was realized based on faecal collection, we did not handle or disturb animals during the study period. No other approval was thus required. Methods developed in this study were carried out in accordance with to the relevant guidelines and regulations.

## Results

### Overall diversity of *Entamoeba* OTUs

**Figure 2:**
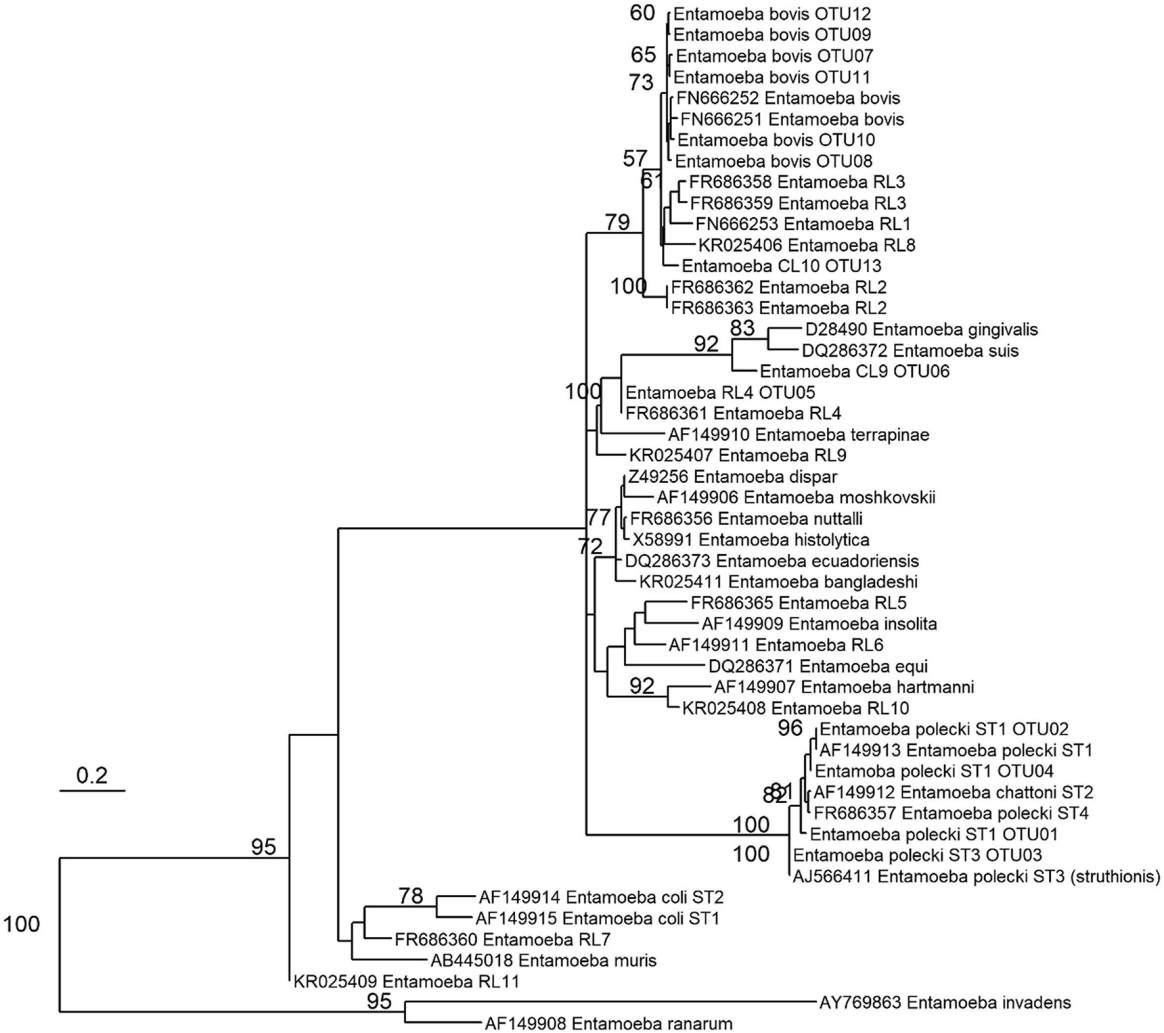
Phylogenetic relationships of *Entamoeba* species based on 292 total positions in the *18S rDNA* gene. Tree was inferred using an unrooted maximum likelihood method. The evolutionary distances were computed using the General Time-Reversible Model modeled using a gamma distribution (shape parameter = 0.5). Bootstrap proportions > 50% (over 1000 replicates) are shown next to the branch nodes. Accession numbers for the reference sequences are listed behind the *Entamoeba* species name. The scale bar represents 0.2 substitution per site.

After applying all bioinformatics filtering steps, the resulting datasets included 643,401 sequences for the run comprising only samples from Yunnan snub-nosed monkey and 731,999 sequences for the run comprising samples from domesticated animals and humans. Thirteen OTUs were detected in 65 of the 115 faeces tested (all hosts combined), all being assigned to *Entamoeba* spp. Based on phylogenetic relatedness with *Entamoeba* sequences previously published (see Table 2 and Fig. 2), we assigned four and six OTUs to *E. polecki* (OTU01–OTU04) and *E. bovis* (OTU07–OTU12), respectively. OTU05 shared 100% identity with *Entamoeba* RL4 (Table 1) and was thus assigned to this ribosomal lineage. OTU06 and OTU13 were putatively assigned to new conditional lineages: OTU06 shared 97% identity with the closest *Entamoeba* species, *Entamoeba* RL4 (MN749981), isolated in horses, and was designated as CL9. OTU13 shared 95% identity with *E. bovis* (FN666252) and was close to sequences that Jacob et al.^28^ referred as “*E. bovis* and related lineages” common in cattle and some non-human primate species; this OTU was designated as CL10. OTUs that did not share 100% identity with available sequences were deposited on Genbank, accession numbers are indicated (Table 2).

**Table 2:** Taxonomic assignment of OTUs (~ 260pb) detected in 65 of 115 faecal samples.

Taxonomic assignment		OTU	Frequency	GenBank acc nb	Closest sequence			
					Identity (% nt)	GenBank acc nb	Host species	References
*Entamoeba polecki*	ST1	OTU01	51/115	MW718195	98% (253/259)	AF149913 (*E. polecki* ST1)	*Sus domesticus*	^63^
	ST1	OTU02	32/115	–	100% (259/259)	AF149913 (*E. polecki* ST1)	*Sus domesticus*	^63^
	ST3	OTU03	33/115	–	100% (259/259)	AJ566411 (*E. polecki* ST3)	*Struthio camelus*	^64^
	ST1	OTU04	35/115	–	100% (259/259)	LC082305 (*E. polecki* ST1)	*Sus domesticus*	^36^
*Entamoeba* RL4		OTU 05	19/115	–	100% (261/261)	FR686361 (*Entamoeba RL4*)	*Bos taurus*	^56^
*Entamoeba* CL9		OTU 06	4/115	MW718196	97% (256/263)	MN749981 (*Entamoeba* RL4)	*Equus sp.*	^65^
*Entamoeba bovis*		OTU 07	6/115	MW718197	99% (261/262)	LC329317 (*E. bovis*)	*Bos taurus*	^59^
		OTU 08	5/115	–	100% (262/262)	LC329314 (*E. bovis*)	*Bos taurus*	^59^
		OTU 09	4/115	MW718198	99% (260/262)	LC329311 (*E. bovis*)	*Bos taurus*	^59^
		OTU 10	3/115	MW718199	99% (259/262)	FN666252 (*E. bovis*)	*Bos taurus*	^66^
		OTU 11	3/115	MW718200	99% (260/262)	LC329311 (*E. bovis*)	*Bos taurus*	^59^
		OTU 12	2/115	MW718201	99% (260/262)	LC329311 (*E. bovis*)	N/A (soil)	^59^
*Entamoeba* CL10		OTU 13	3/115	MW718202	95% (248/262)	FN666252 (*E. bovis*)	*Bos taurus*	^66^

Frequency of each OTU is given all hosts combined. For each OTU, information on the closest sequence (through BLASTn searches) listed in reference sequences comprise: identity of the sequence, GenBank accession number, host species from which the sequence was amplified for the first time, and bibliographic reference associated to the sequence.

**Figure 3:**
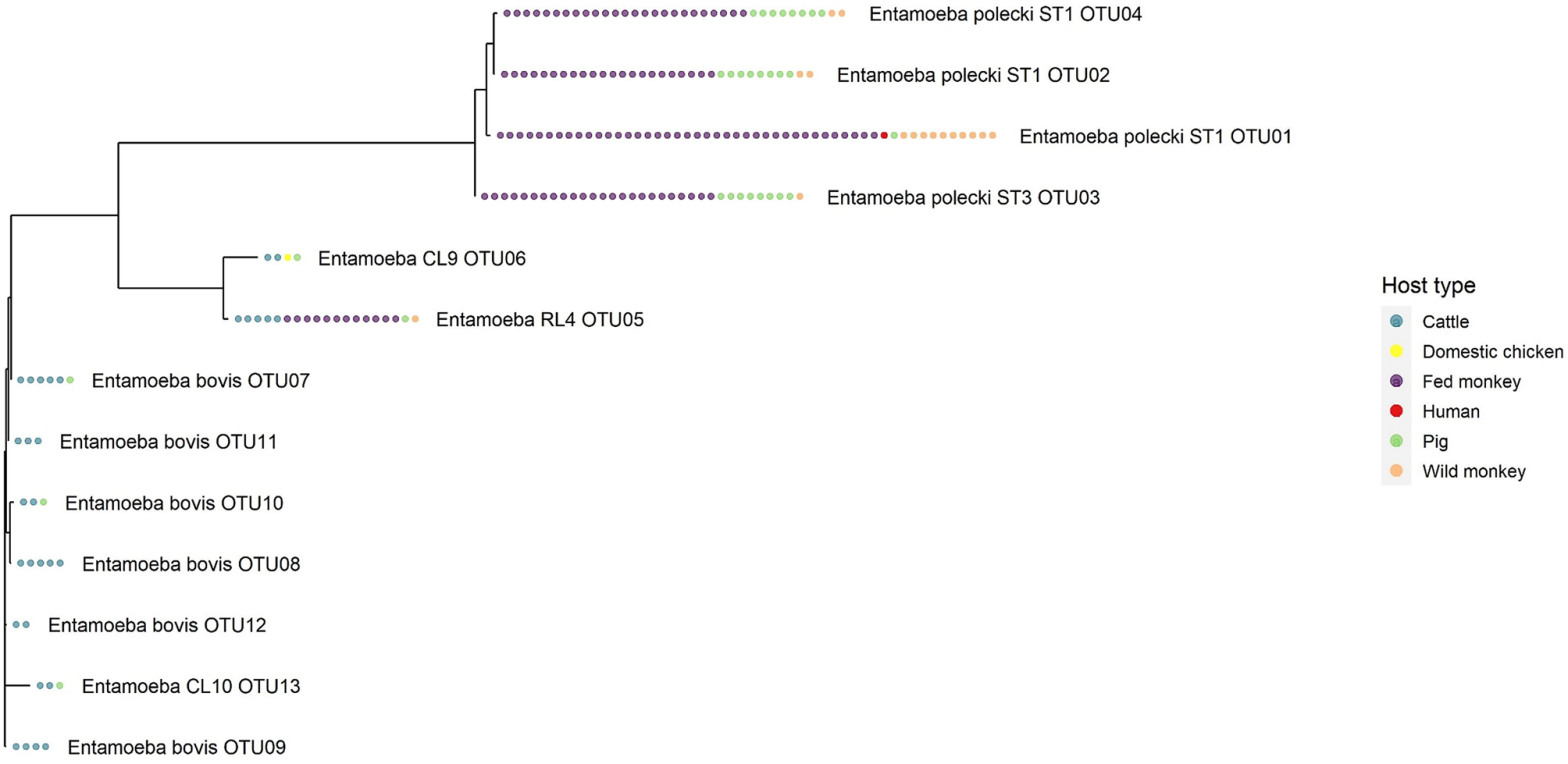
Host spectrum and number of positive faecal samples for all the 13 *Entamoeba* OTUs amplified in 65 of 115 faecal samples.

The phylogenetic relationships of the OTUs amplified in this study suggest three clusters in our data, which coincided with different host profiles (Fig. 3): (1) an *E. polecki* cluster, with four OTUs amplified in faeces of monkeys, humans, and pigs, (2) a cluster comprising *Entamoeba* RL4 and *Entamoeba* CL9, amplified in faeces of cattle, monkeys, pigs and domestic chickens, and (3) a cluster of *E. bovis* and the related conditional lineage CL10 amplified only in faeces of cattle and pigs.

### Proportion of faecal samples PCR-positive for *Entamoeba*

**Figure 4:**
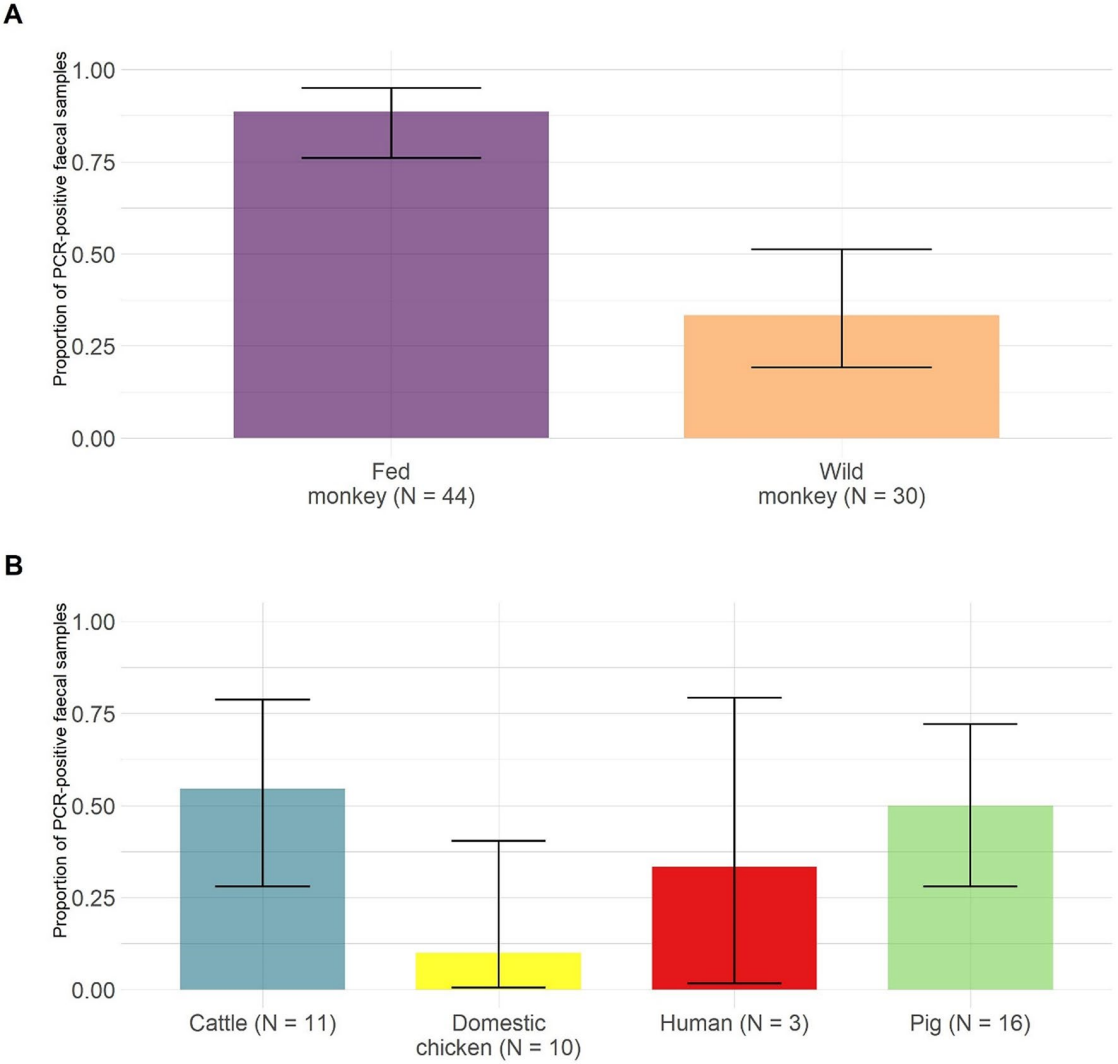
Proportion of faecal samples PCR-positive for at least one *Entamoeba* OTU in the different hosts: (**A**) in Yunnan snub-nosed monkeys, individual prevalence in faecal samples was assessed using individual microsatellite genotyping, (**B**) in other hosts, faecal prevalence was assessed without individual identification. Numbers in brackets represent sample sizes.

**Figure 5:**
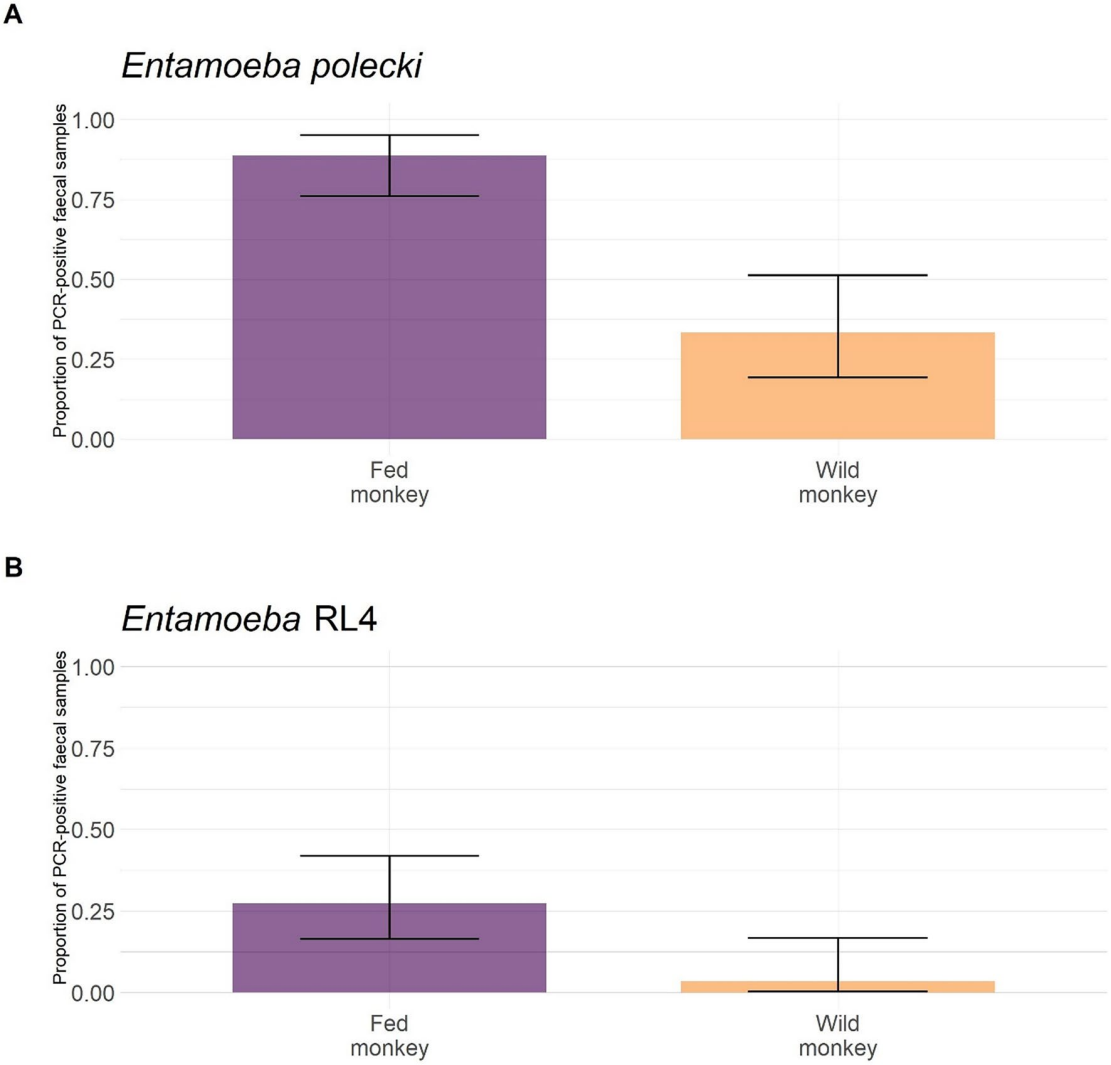
*Entamoeba* faecal prevalence (i.e. proportion of individuals with at least one *Entamoeba* OTU detected by PCR) by species or lineages in Yunnan snub-nosed monkeys.

The proportion of individual that had a faecal sample PCR-positive for at least one *Entamoeba* OTU was significantly higher in fed monkeys (0.89, 95%CI 0.76–0.95; Fig. 4A) than in wild monkeys (0.33, 95%CI 0.19–0.51; χ^2^ = 21.98, df = 1, *P* < 0.001). We did not detect autocorrelation due to sampling date in PCR-positivity neither in fed (DW = 2.27, *P* = 0.780) nor in wild monkeys (DW = 2.21, *P* = 0.678). *Entamoeba polecki* and *Entamoeba* RL4 were the only species/lineages found in monkeys, with different prevalence between fed and wild monkeys (Fig. 5): prevalence for *E. polecki* and *Entamoeba* RL4 was both higher in fed than wild monkeys (χ^2^ = 21.98, df = 1, *P* < 0.001 and χ^2^ = 5.50, df = 1, *P* = 0.019, respectively).

**Figure 6:**
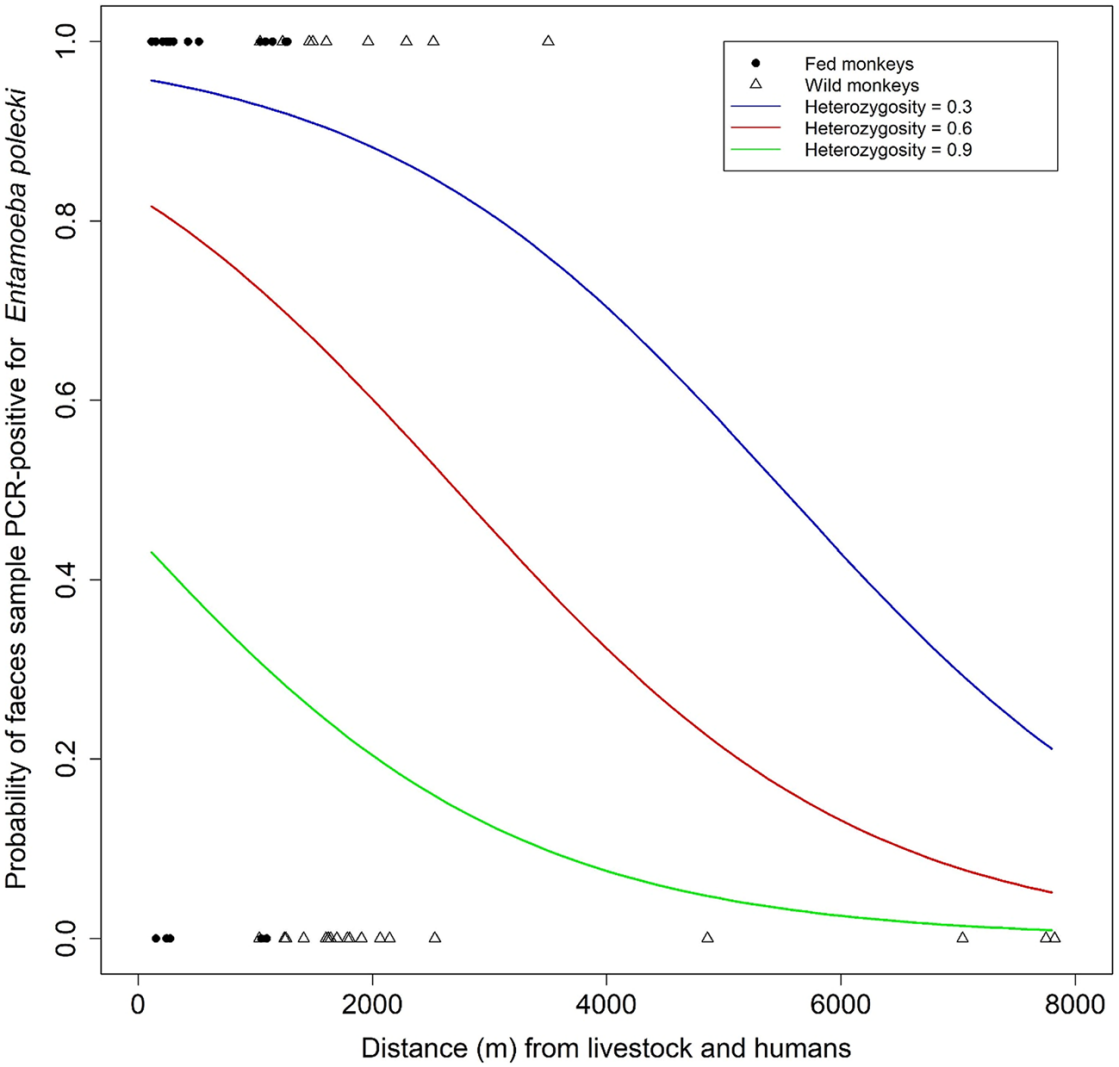
Model predictions for faecal samples positivity for *Entamoeba polecki* OTUs in 74 Yunnan snub-nosed monkeys. Probability for faecal sample to be PCR-positive for at least one *Entamoeba polecki* OTU in function of distance to the centroid of faecal sample locations recorded in humans and livestock, and to monkey individual heterozygosity observed over 10 microsatellites. Values of individual heterozygosity were fixed on the mean value (0.6) and the 95 percentile values of heterozygosity (0.3 and 0.9).

**Figure 7:**
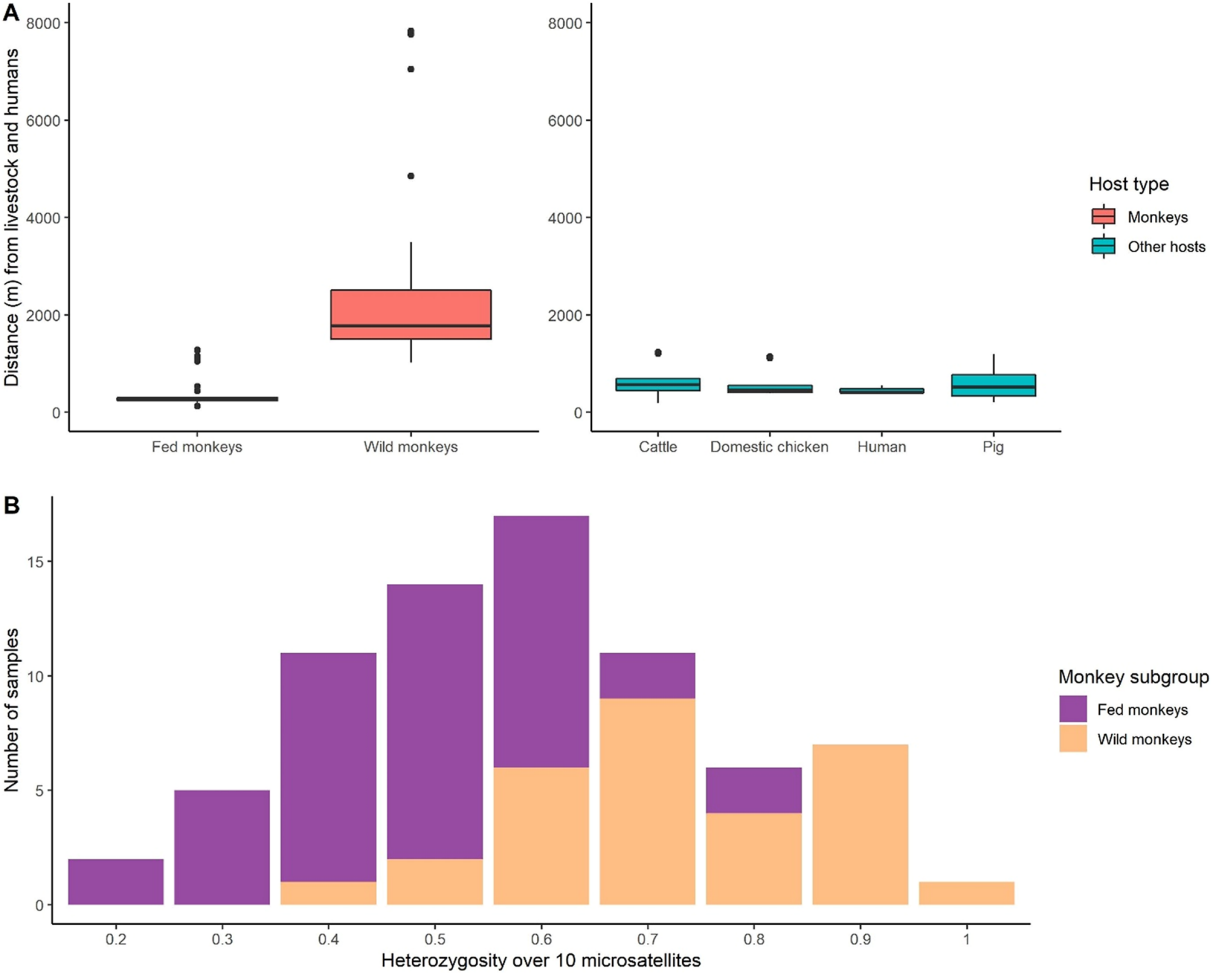
(**A**) Boxplot of distances (in meters) of faecal samples from the centroid of faecal sample locations recorded in livestock and humans. (**B**) Distribution of heterozygosity in Yunnan snub-nosed monkeys averaged on ten microsatellites.

The model with the lowest AIC showed that the probability that a given individual monkey had a faecal sample PCR-positive for at least one *E. polecki* OTU was related to both the distance from the centroid of faecal sample locations of livestock and humans (ΔAICc = 19.01, β = − 0.0006, SE_β_ = 0.00003, LRT *P* < 0.001), and individual heterozygosity in monkeys (ΔAICc = 5.53, β = − 5.62, SE_β_ = 2.21, LRT *P* < 0.001; see detailed results on model selection Table 1). The subgroup within which monkeys were distributed (fed or wild) was not related to faecal sample positivity after taking into account these two variables (ΔAICc = 0.49). Probability rapidly decreased with distance from livestock and humans to reach only negative samples after 4000 m, and probability decreased when heterozygosity increased (Fig. 6). The highest risk of faecal positivity in monkeys was thus reached when individuals were close to livestock and humans, and had a low individual heterozygosity. These two criteria are likely to be found mainly in fed monkeys, for which the lowest values of distance and heterozygosity were recorded (Fig. 7). Conversely, individual monkeys far from livestock and humans and with high heterozygosity were mainly recorded in the wild subgroup (Fig. 7) and were predicted to have a very low probability of *E. polecki* PCR-positivity in their faeces.

**Figure 8:**
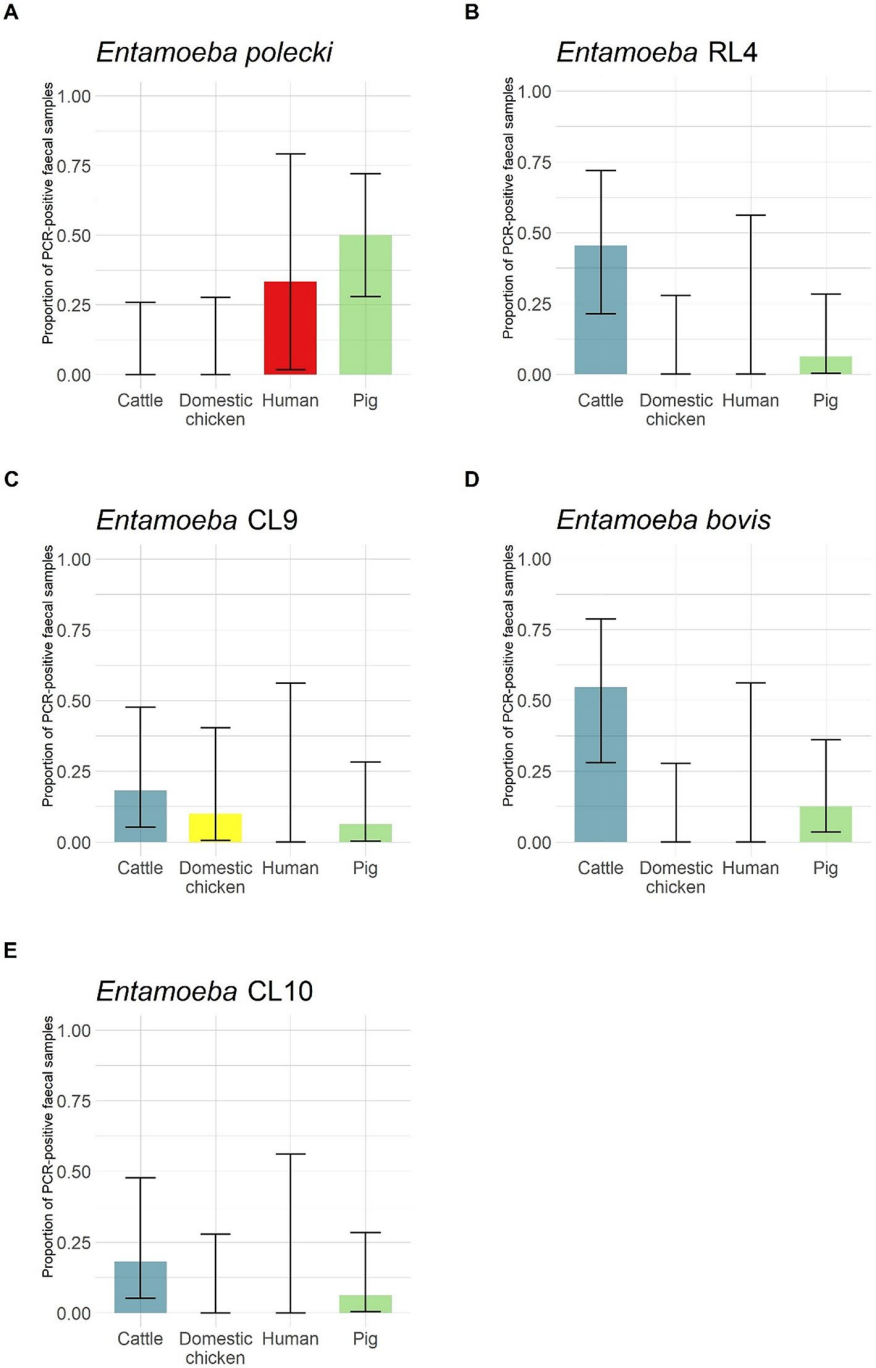
*Entamoeba* faecal prevalence (i.e. proportion of faecal samples with at least one *Entamoeba* OTU detected by PCR) by species or lineages in livestock and humans.

*Entamoeba polecki* was the only species detected in humans in one faecal sample (Fig. 8A), while *Entamoeba* CL9 was the only species detected in domestic chickens (1/10 faecal sample; Fig. 8C). Pigs and cattle were more frequently PCR-positive for *Entamoeba* (Fig. 4B). All species/lineages were found in pig faeces (Fig. 8), with *E. polecki* being predominant in this host. Conversely, *E. polecki* was not found in cattle faeces, in which *Entamoeba* RL4 and *E. bovis* were frequently found (Fig. 8).

### *Entamoeba* OTU co-occurrences and assemblages in hosts

**Figure 9:**
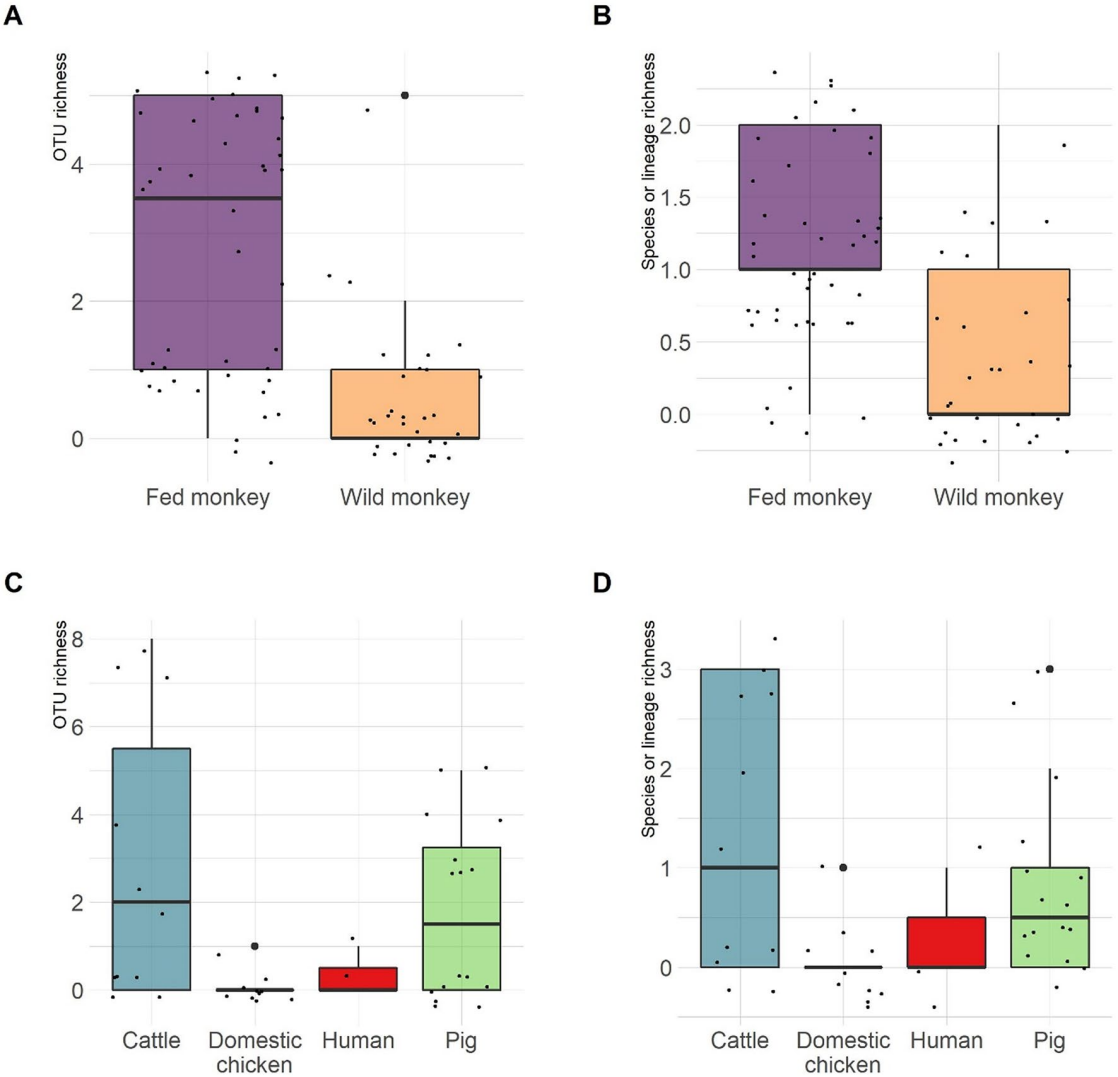
Boxplot of number of (**A**) *Entamoeba* OTU and (**B**) *Entamoeba* species or lineages detected in faecal samples of 74 Yunnan snub-nosed individual monkeys, and (**C**) *Entamoeba* OTU and (**D**) *Entamoeba* species or lineages detected in faecal samples of livestock and humans.

Several *Entamoeba* OTUs frequently co-occurred in faecal samples. The co-occurence of more than one *Entamoeba* OTU in PCR-positive samples was higher in in fed monkeys (88.64%) than in wild monkeys (33%), and was observed in 54.5% of cattle, and 50% of pigs. OTU richness was higher in fed than wild monkeys (Fig. 9A; Fisher’s exact test for count data, *P* < 0.001) and was 2.77 in fed monkeys and 0.53 in wild monkeys on average. Consequently, co-occurrence of *Entamoeba* species/lineages was observed more frequently in fed than wild individuals (Fig. 9B). *Entamoeba* OTU diversity in positive samples (i.e. with at least one *Entamoeba* OTU) did not significantly differed between fed and wild monkeys (PERMANOVA, F = 2.15, df = 1 and 47, *P* = 0.121). Co-occurrences were frequently observed in cattle and pigs, regarding both OTU richness and *Entamoeba* species/lineages (Fig. 9C,D).

**Figure 10:**
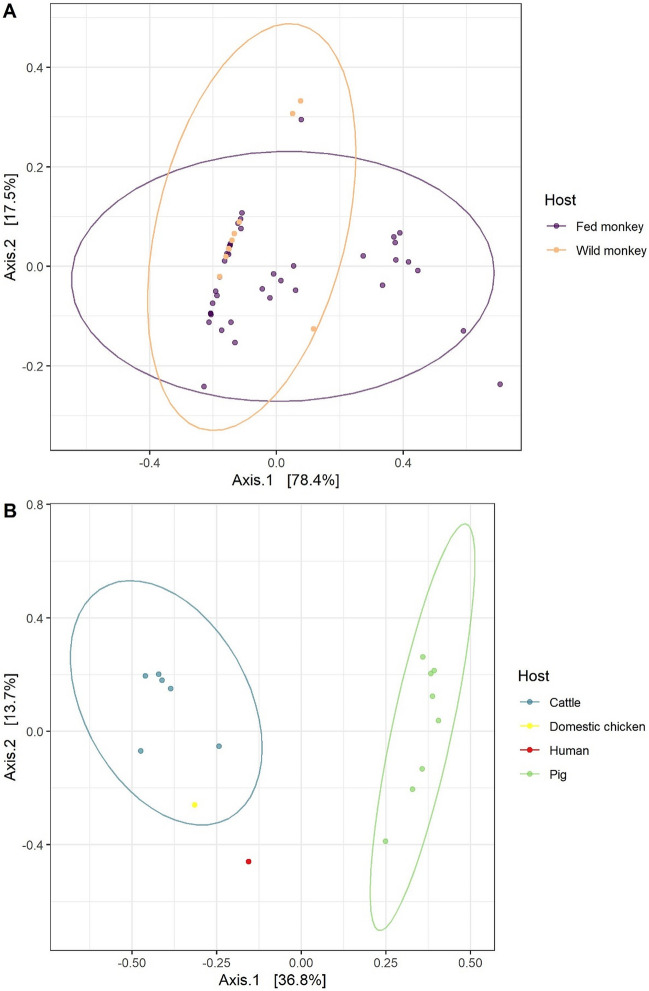
Principal Coordinate Analysis ordination for Bray–Curtis dissimilarities of *Entamoeba* OTU assemblages.

PCoA analysis confirmed that *Entamoeba* OTU assemblages overlapped in fed and wild monkeys (Fig. 10A): wild monkeys showed assemblages similar to some of the OTU profiles observed in fed monkeys. Cattle (and one PCR-positive faeces of domestic chicken) formed a group relatively differentiated from pigs (Fig. 10B).

## Discussion

In this study, we took advantage of the recent development of high-throughput sequencing for the PCR-diagnosis of *Entamoeba* to investigate parasitism in a local host assemblage strongly influenced by wildlife-related tourism via food provisioning. The 13 *Entamoeba* OTUs found in this study were distributed in three phylogenetic clusters, no one of these clusters being specific to a given host. We assigned these OTUs to *E. polecki*, *Entamoeba* RL4, *E. bovis*-related lineages, and we named two conditional lineages (CL9 and CL10) that have not been yet reported.

In Yunnan snub-nosed monkeys, the individual faecal prevalence of *Entamoeba* OTUs was higher in fed (89%) than in wild monkeys (33%). We also found co-occurences of *Entamoeba* OTUs more frequently in the fed subgroup, all these OTUs being assigned to *E. polecki* or *Entamoeba* RL4. These elements suggest that fed individuals face a higher exposure to parasite transmission than wild individuals. While *E. polecki* is commonly reported in non-human primates, *Entamoeba* RL4 has only been found in cattle previously^28,55^. Our study is to our knowledge the first report of this lineage in faeces of non-human primates and pigs. One possible explanation is that Yunnan snub-nosed monkey and pigs might carry the parasite after ingestion from an environment contaminated by cattle droppings. The fact that cysts of several *Entamoeba* species were detected in environmental samples (soil and water) in previous studies^58^ supports this hypothesis. It is also possible that monkeys and pigs ingest other host faeces (particularly from cattle grazing in feeding sites), either accidentally or on purpose. However, DNA amplified in host faeces might actually originate from soil and have contaminated faeces before sampling collection. If we assume that Yunnan snub-nosed monkeys or pigs carried the parasite in their digestive tract, we also do not know if they are a natural host for *Entamoeba* RL4 or if they are transient hosts.

In Yunnan snub-nosed monkeys, the probability for a faecal sample to be PCR-positive for at least one *E. polecki* OTU was related to the distance from livestock and humans, and to monkey individual multilocus heterozygosity. This probability was the highest when faecal samples were collected close to livestock and humans, and when individual heterozygosity was low. Multilocus heterozygosity has already been related to parasite infection likelihood in a variety of hosts and parasites^38^. Although highly debated, multilocus heterozygosity is believed to be linked to individual fitness^59,60^. In our case, fed monkeys show low genetic diversity and high relatedness^26^, which would theoretically lead to decreased chances of surviving disease. However, the distribution of individual heterozygosity overlaps with a gradient of domesticated animal and human density. Our sampling design does not allow to sort out this covariation, and further investigations should be conducted to determine if there is a higher risk of diseases in the fed subgroup due to fitness variability.

**Figure 11:**
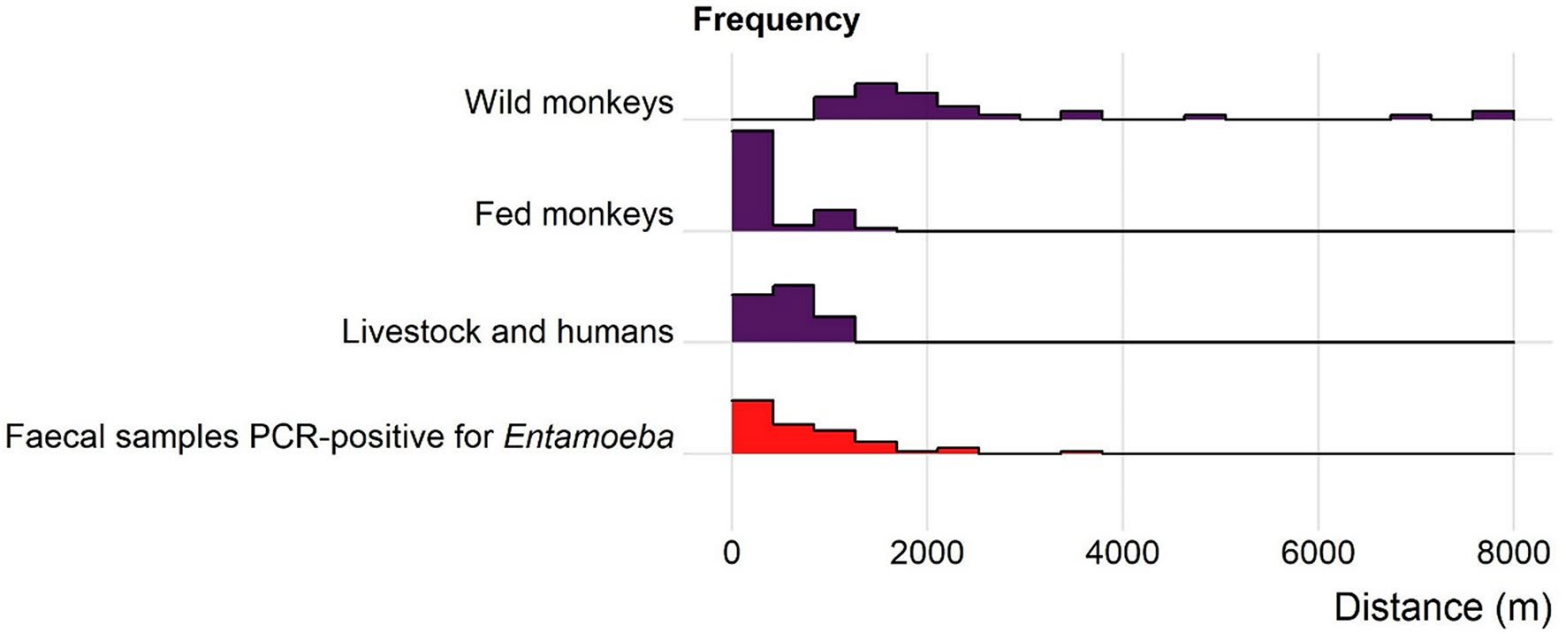
Frequency of faecal samples and *Entamoeba* PCR-positivity as a function of distance to the centroid of locations where livestock and human faeces were collected.

Our main hypothesis to explain the high prevalence for *Entamoeba* observed in the fed subgroup is that host aggregation at feeding sites can promote parasite transmission, and increase inter-specific contacts. Rather than study the distance to feeding sites, we focused on the distance to livestock and human settlements and found here a proxy which might be used to determine a reasonable distance between anthropized areas and places where feeding sites can be implemented (Fig. 11). We believe that if this measure is repeated in other systems, it can help managers to mitigate the bidirectional risk of disease transmission due to a wild-domestic interface. Here, we found that the probability of finding a faeces positive for *E. polecki* in monkeys rapidly decreased with the distance from livestock and humans. Faeces of wild monkeys are often difficult to collect, due to evasiveness in large and scarped mountain, and we probably do not have enough data to support an empirical cut-off value. Nevertheless, the few faeces from wild monkeys collected over 4 km from livestock and humans were all *Entamoeba* free.

 In this study, *E. polecki* was found in faeces of Yunnan snub-nosed monkeys, pigs, and humans. These results are consistent with previous studies which all concluded that *E. polecki* ST1 to ST4 are not host-specific and are generally found in these hosts^28,35^. *Entamoeba polecki* (also refered as *E. chattoni* in non-human primates) is commonly found in non-human primates, especially in Asia, where it is not rare to observe high prevalence^27,61,62^. *Entamoeba polecki* is also a common parasite of domesticated pigs^28^. Here, we show evidences that humans, domesticated and wild animals can all be exposed to the same parasite. However, the fact that some habituated individuals of the endangered Yunnan snub-nosed monkey were highly exposed to *Entamoeba*, including an *Entamoeba* species also found in humans and domesticated animals raises some questions for the conservation of this group of monkeys. Even if the *Entamoeba* species found in this study have not been related to infectious diseases in hosts, the pathogenicity of *Entamoeba* species is largely unknown and has never been explored in the case of the Yunnan snub-nosed monkey. Moreover, if we assume that feeding sites overlapping livestock distribution ranges are local hotspots of interspecific parasite transmission, parasite exposure for these monkeys may concern a wide range of parasites (including bacteria, viruses, helminths, and other protozoa). To some extent, our study is a model of how contacts between wildlife and domestic animals and humans reinforce interspecific exchange of parasitic organisms. As distance to other hosts seems to be a determinant of positivity for parasites, we recommend avoiding overlapping grazing areas and feeding sites. More generally, a systemic Ecohealth approach should be considered to ensure both conservation of Yunnan snub-nosed monkey and the health of human and domesticated animals, which are inseparable in this social–ecological system.

## Supplementary Information

Supplementary Information.

## Data Availability

Supplementary data are available on Zenodo (10.5281/zenodo.5137214): (i) raw sequencing reads, (ii) operational taxonomic unit (OTU) table, (iii) sample metadata, (iv) OTU DNA sequences and (v) their taxonomic assignations.
