# Supplementary material for: Feeding sites promoting wildlife-related tourism might highly expose the endangered Yunnan snub-nosed monkey (Rhinopithecus bieti) to parasite transmission
Source: Sci Rep. 2021 Aug 4;11:15817. doi: 10.1038/s41598-021-95166-5 (PMC8339071; doi:10.1038/s41598-021-95166-5)
Supplement: Supplementary file 1 — Supplementary Information. [file 41598_2021_95166_MOESM1_ESM.docx]

**Supplementary material for**

**Feeding sites promoting wildlife-related tourism might highly expose the endangered Yunnan snub-nosed monkey (Rhinopithecus bieti) to parasite transmission**

Eve Afonso^1,*^, Rong Fu^1,2^, Amaël Dupaix^1,3^, Anne-Claude Goydadin^1^, ZhongHua Yu^4^, Cécile Callou^5^, Petra Villette^1^, Patrick Giraudoux^1,2,§^, Li Li^2,§^

^1^ UMR CNRS 6249 Chrono-environnement, Université Bourgogne Franche-Comté, 16 Route de Gray, 25030 Besancon, France

^2^Laboratory of Wildlife Management and Ecosystem Health, Yunnan University of Finance and Economics, Kunming, China

^3^Département de Biologie, ENS Lyon, 46 allée d’Italie, 69007 Lyon, France

^4^National Nature Reserve of BaiMa XueShan, Tacheng, China

^5^Muséum national d’Histoire naturelle, UMR 7209 du CNRS, Archéozoologie, Archéobotanique : sociétés, pratiques et environnements, case postale 55, 55 rue Buffon, 75005 Paris, France

**^*^** Corresponding author

Phone number: +33 3 81 66 57 91

E-mail address: eve.afonso@univ-fcomte.fr

**S1. Protocol for the microsatellite genotyping of Yunnan snub-nosed monkeys**

Genotyping was carried out by amplifying 10 microsatellite markers published by Hao et al. (2007) and Liu et al. (2008). Each reaction was performed in a mixture (14 µL) consisting of 50-100 ng of DNA extract, 1× Multiplex PCR Master Mix (Qiagen), and primer concentrations as reported in Table S1.1. The amplification programme comprised an activation step of 15 min at 95°C, followed by 40 cycles of denaturation at 94°C for 30 s, annealing at 52°C or 58°C (see Table S1.1) for 90 s, and primer extension at 72°C for 60 s. A final extension was performed for 30 min at 68°C. The absence of cross-contamination was confirmed by including PCR negative controls (including PCR-grade water instead of DNA extract). PCR products were mixed in three panels as described Table S1.1. Allele size was quantified using an Applied Biosystems 3130 Genetic Analyzer. To score alleles, we used the package Fragman (Covarrubias-Pazaran et al. 2016) in R 3.5.1 software (R Development Core Team. R: A Language and Environment for Statistical Computing, R Foundation for Statistical Computing, Vienna, Austria, 2018. <http://www.r-project.org>).

To reduce genotyping errors, we used a comparative multiple-tube approach to determine multilocus genotypes as recommended by Taberlet et al. (1996). We used the allelematch package (Galpern et al. 2012) in R software to perform multiple comparisons between genetic profiles.

**Table S1.1.** **Microsatellites used for the genotyping of the Yunnan snub-nosed monkey (*Rhinopithecus bieti*) from faeces samples:** forward (F) and reverse (R) primer sequence for each locus, size range of PCR products observed in this study, number of alleles amplified for each locus, concentration of primers in PCR, annealing temperature (Ta) in the PCR programme, coloured dye and references. The PCR products were regrouped in three panels (panel 1, 2 and 3) for the allele scoring step. PCR products were grouped in three panels before fragment length analysis.

| **Locus** | **Primer sequence (5’ – 3’)** | **Size range (bp)** | **No. of alleles** | **Cc° (µM)** | **Ta (°C)** | **Dye** | **Reference** |
| --- | --- | --- | --- | --- | --- | --- | --- |
| **Panel 1** |  |  |  |  |  |  |  |
| GM108 | F:CAGCGTAAGCCAGTTGCC  R:GGAAAAGTCTGAAACCCACGA | 121-135 | 8 | 0.27 | 58 | 6-FAM | (Hao et al. 2007) |
| GM109 | F:GGTGGAGGAGGGCCTAAC  R:CTGATGTCCATAGGCGACCAT | 134-162 | 7 | 0.27 | 58 | JOE | (Hao et al. 2007) |
| GM209 | F:ATCTGAATGATGTGTGGATGT  R:TAGAGTAGCATTGCCT | 144-164 | 9 | 0.32 | 58 | ROX | (Hao et al. 2007) |
| **Panel 2** |  |  |  |  |  |  |  |
| D6S493 | F: ATCCCAACTCTTAAATGGGC  R: TTCCATGGCAGAAATTGTTT | 254-270 | 8 | 0.32 | 58 | 6-FAM | (Liu et al. 2008) |
| D8S505 | F: CAAAAGTGAACCCAAACCTA  R: AGTGCTAAGTCCCAGACCAA | 146-160 | 8 | 0.43 | 58 | 6-FAM | (Liu et al. 2008) |
| D17S1290 | F: GCCAACAGAGCAAGACTGTC  R: GGAAACAGTTAAATGGCCAA | 222-240 | 8 | 0.32 | 58 | ROX | (Liu et al. 2008) |
| GM214 | F:GGGCAACAGAGCGAGACTG  R:TGCAAAGATGTGAACGGAAAT | 138-156 | 8 | 0.27 | 58 | JOE | (Hao et al. 2007) |
| **Panel 3** |  |  |  |  |  |  |  |
| D1S533 | F:CATCCCCCCCAAAAAATATA  R:TTGCTAATCAAATAACAATGGG | 205-217 | 6 | 0.27 | 52 | ROX | (Liu et al. 2008) |
| D5S1457 | F: TAGGTTCTGGGCATGTCTGT  R: TGCTTGGCACACTTCAGG | 117-141 | 12 | 0.32 | 52 | JOE | (Liu et al. 2008) |
| D20S206 | F: TCCATTATTCCCCTCAAACA  R: GGTTTGCCATTCAGTTGAGA | 116-132 | 7 | 0.43 | 52 | 6-FAM | (Liu et al. 2008) |

**S2. Dual-indexing primers for 18S rRNA sequencing (673f/942r) of *Entamoeba* spp. on the MiSeq Platform**

**1. 673f (forward primer) PCR amplification primer sequence (after (Fadrosh et al. 2014)):**

Field (space-delimited), description

1, 5’ Illumina Linker Sequence

2, Index 1

3, Heterogeneity Spacer

4, 673f Primer Sequence (after (Vlčková et al. 2018)

CAAGCAGAAGACGGCATACGAGATGTGACTGGAGTTCAGACGTGTGCTCTTCCGATCT CCTAAACTACGG ATYAGATACCGTCGTAGTCC

CAAGCAGAAGACGGCATACGAGATGTGACTGGAGTTCAGACGTGTGCTCTTCCGATCT TGCAGATCCAAC ATYAGATACCGTCGTAGTCC

CAAGCAGAAGACGGCATACGAGATGTGACTGGAGTTCAGACGTGTGCTCTTCCGATCT CCATCACATAGG ATYAGATACCGTCGTAGTCC

CAAGCAGAAGACGGCATACGAGATGTGACTGGAGTTCAGACGTGTGCTCTTCCGATCT GTGGTATGGGAG T ATYAGATACCGTCGTAGTCC

CAAGCAGAAGACGGCATACGAGATGTGACTGGAGTTCAGACGTGTGCTCTTCCGATCT ACTTTAAGGGTG T ATYAGATACCGTCGTAGTCC

CAAGCAGAAGACGGCATACGAGATGTGACTGGAGTTCAGACGTGTGCTCTTCCGATCT GAGCAACATCCT T ATYAGATACCGTCGTAGTCC

CAAGCAGAAGACGGCATACGAGATGTGACTGGAGTTCAGACGTGTGCTCTTCCGATCT TGTTGCGTTTCT GT ATYAGATACCGTCGTAGTCC

CAAGCAGAAGACGGCATACGAGATGTGACTGGAGTTCAGACGTGTGCTCTTCCGATCT ATGTCCGACCAA GT ATYAGATACCGTCGTAGTCC

CAAGCAGAAGACGGCATACGAGATGTGACTGGAGTTCAGACGTGTGCTCTTCCGATCT AGGTACGCAATT GT ATYAGATACCGTCGTAGTCC

CAAGCAGAAGACGGCATACGAGATGTGACTGGAGTTCAGACGTGTGCTCTTCCGATCT ACAGCCACCCAT CGA ATYAGATACCGTCGTAGTCC

CAAGCAGAAGACGGCATACGAGATGTGACTGGAGTTCAGACGTGTGCTCTTCCGATCT TGTCTCGCAAGC CGA ATYAGATACCGTCGTAGTCC

CAAGCAGAAGACGGCATACGAGATGTGACTGGAGTTCAGACGTGTGCTCTTCCGATCT GAGGAGTAAAGC CGA ATYAGATACCGTCGTAGTCC

**2. 942r (forward primer) PCR amplification primer sequence (after (Fadrosh et al. 2014)):**

Field (space-delimited), description

1, 3’ Illumina Linker Sequence

2, Index 2

3, Heterogeneity Spacer

4, 942r Primer Sequence (Vlčková et al. 2018)

AATGATACGGCGACCACCGAGATCTACACTCTTTCCCTACACGACGCTCTTCCGATCT CCTAAACTACGG GTWCGGTCTTGGTAAGTTTTC

AATGATACGGCGACCACCGAGATCTACACTCTTTCCCTACACGACGCTCTTCCGATCT TGCAGATCCAAC GTWCGGTCTTGGTAAGTTTTC

AATGATACGGCGACCACCGAGATCTACACTCTTTCCCTACACGACGCTCTTCCGATCT CCATCACATAGG GTWCGGTCTTGGTAAGTTTTC

AATGATACGGCGACCACCGAGATCTACACTCTTTCCCTACACGACGCTCTTCCGATCT GTGGTATGGGAG A GTWCGGTCTTGGTAAGTTTTC

AATGATACGGCGACCACCGAGATCTACACTCTTTCCCTACACGACGCTCTTCCGATCT ACTTTAAGGGTG A GTWCGGTCTTGGTAAGTTTTC

AATGATACGGCGACCACCGAGATCTACACTCTTTCCCTACACGACGCTCTTCCGATCT GAGCAACATCCT A GTWCGGTCTTGGTAAGTTTTC

AATGATACGGCGACCACCGAGATCTACACTCTTTCCCTACACGACGCTCTTCCGATCT TGTTGCGTTTCT TC GTWCGGTCTTGGTAAGTTTTC

AATGATACGGCGACCACCGAGATCTACACTCTTTCCCTACACGACGCTCTTCCGATCT ATGTCCGACCAA TC GTWCGGTCTTGGTAAGTTTTC

**References**

Fadrosh, Douglas W., Bing Ma, Pawel Gajer, et al.
 2014 An Improved Dual-Indexing Approach for Multiplexed 16S RRNA Gene Sequencing on the Illumina MiSeq Platform. Microbiome 2(1): 6.

Hao, Y. L., Z. J. Liu, H. Wu, et al.
 2007 Isolation and Characterization of 11 Microsatellite Loci for the Sichuan Snub-Nosed Monkey, Rhinopithecus Roxellana. Conservation Genetics 8(5): 1021–1024.

Liu, Z. J., B. P. Ren, Y. L. Hao, et al.
 2008 Identification of 13 Human Microsatellite Markers via Cross-Species Amplification of Fecal Samples from Rhinopithecus Bieti. International Journal of Primatology 29(1): 265–272.

Vlčková, Klára, Jakub Kreisinger, Barbora Pafčo, et al.
 2018 Diversity of Entamoeba Spp. in African Great Apes and Humans: An Insight from Illumina MiSeq High-Throughput Sequencing. International Journal for Parasitology 48(7): 519–530.
